# Development and evaluation of taste masked dry syrup formulation of potassium chloride

**DOI:** 10.1186/s41120-019-0030-z

**Published:** 2019-01-22

**Authors:** Madhur Kulkarni, Brijesh Vishwakarma, Samik Sen, Sandhya Anupuram, Abhijit A. Date

**Affiliations:** 1 Department of Pharmaceutics, SCES’s Indira College of Pharmacy, 89/2a, Niramay, New Mumbai Pune Highway, Tathawade, Pune, Maharashtra 411033 India; 2 Gansons Ltd, Kolshet Road, Manpada, Thane, Maharashtra 400607 India; 3 Department of Pharmaceutical Sciences, The Daniel K. Inouye College of Pharmacy, University of Hawaii Hilo, 200 W. Kawili Street, Hilo, HI 96720 USA

**Keywords:** Taste masking, KCl, Eudragit E100, Fluid bed processing, Electronic taste sensing machine

## Abstract

Potassium chloride (KCl) syrup is widely used for the oral treatment of the hypokalemia. However, it is associated with unacceptable taste. In the present study, we sought to develop a palatable and easy to reconstitute KCl dry syrup as a commercially viable alternative to currently available KCl syrup. We explored the potential of Eudragit E100 as a taste-masking polymer to coat and improve the palatability of the KCl. With the help of fluid bed processor, KCl was coated with the solution containing varying amounts of Eudragit E100 (4, 6, 10 and 15%). Coating with 10% polymer solution enabled optimal fluid bed processing, higher entrapment of the KCl (81%) and better in vitro release profile in 0.1 N HCl and pH 6.8 phosphate buffer. A dry syrup formulation containing Eudragit E100 coated KCl with good physical and chemical stability in dry and reconstituted state was developed. The palatability of the optimized formulation and commercially available KCl syrup was evaluated using the Electronic Taste Sensing Machine. The developed formulation showed~ 2-fold better taste-masking compared to the commercial KCl syrup. Thus, present investigation describes the development of an effective alternative to the current KCl syrup that can offer better palatability, stability and patient compliance.

## Introduction

Hypokalemia is characterized by the potassium depletion from the body. Patients receiving diuretics treatment, those suffering from diabetic ketoacidosis or primary and secondary hyperaldosteronism often experience hypokalemia (Cohn et al., 2000). Potassium depletion due to these causes is usually accompanied by a concomitant loss of chloride. Potassium chloride (KCl) is an electrolyte of choice for the treatment of hypokalemia as it can replenish potassium as well as chloride ions in the patients experiencing hypokalemia (Shen, 1996; Gennari, 1998). Patients with hypokalemia typically require a dose between 40 and 100 mEq of potassium per day. The US FDA has approved a number of immediate and extended release KCl formulations which contain a relatively high amount of KCl (600–1500 mg) to meet the daily requirement (Mittapalli et al., 2017). Due to a relatively high dose of KCl, these formulations are bulky and are difficult to swallow especially for the paediatric and geriatric population.

Another issue with oral KCl therapy is its very unacceptable taste which often leads to nausea and vomiting. Furthermore, sudden availability of large dose of KCl also leads to gastric irritation which further exacerbates nausea and vomiting (Mcmahon et al., 1982). The film coated tablets and extended release capsule and tablet formulations minimize this side effect (Wu et al., 2003; Graham et al., 1990; Chang and Rudnic, 1991; Kumar et al., 2011). However, these formulations are not very suitable for paediatric and geriatric patients. Furthermore, in India, only KCl syrup and solutions are available to the patients (CIMS, 2017) and these formulations have very unacceptable taste. Here, we report the development of KCl dry syrup with improved palatability. To improve the palatability of KCl, we directly coated pure KCl crystals with Eudragit E 100, a pH sensitive amino alkyl methacrylate copolymer by means of fluid bed processing. This strategy masked the salty taste of KCl and prevented its instantaneous release in the stomach. This simple and commercially viable approach is expected to significantly improve compliance and acceptability of KCl syrup formulations in the patients especially in the paediatric and geriatric population.

## Materials and methods

### Materials

KCl, sucralose and cherry flavour, methyl paraben and propyl paraben were purchased from Analab Fine chem, Ltd., Pune, India. Talc (Luzenac pharma M) and titanium dioxide (Kronos 1171) were purchased from Signet Chemicals, Mumbai, India. Triethyl citrate was purchased from Loba Chemie, Mumbai, India. Silver nitrate (AgNO_3_) was purchased from Merck chemicals, Mumbai. India. Eudragit® E 100 (copolymer of dimethyl aminoethyl methacrylate, butyl methacrylate, and methyl methacrylate) was obtained as a gift sample from Evonik Industries, Mumbai, India. Avicel® CL 611 (Microcrystalline cellulose and carboxy methyl cellulose) was purchased from Hiranya cellulose products, Mumbai, India.

### Fluid bed coating

#### Preparation of coating solution

The coating solution was prepared by dissolving Eudragit E100 in the mixture of isopropyl alcohol and acetone (60:40 ratio). Triethyl citrate (plasticizer) was added and dissolved in the solution of polymer. Talc (anti-adherent) and titanium dioxide (opacifier) were passed through 80# sieve and dispersed slowly into the Eudragit solution under stirring to form a uniform suspension (Table 1). The suspension was stirred for 30 min before beginning the coating process and then throughout the coating to avoid settling of suspended solids.

**Table 1:** Compositions of coating solutions used for fluid bed coating of KCl

Excipient	Composition (%*w*/w)			
	C1	C2	C3	C4
Eudragit E100	4	6	10	15
Talc	0.24	0.36	0.6	0.9
Triethyl citrate	0.14	0.21	0.35	0.52
Titanium dioxide	0.08	0.12	0.2	0.3
Isopropyl alcohol + acetone mixture^a^	q.s. 100	q.s. 100	q.s. 100	q.s. 100

^a^Isopropyl alcohol and acetone mixture was prepared in 60:40 ratio

#### Coating trials

The coating was performed on 400 g batch size of KCl using a fluidized bed Wurster coater (Gansons, India, GFBPC 2 l) inserted with one Wurster column employing bottom spray. The coating procedure involved maintaining the bed temperature at 40 to 43 °C, spray rate of 16 g/min, blower rpm of 800, inlet air pressure of 5.4 Kg/cm^2^, inlet air temperature of 50 °C exhaust temperature of 33 °C and atomizing air pressure in the region of 1.8 to 2.2 bar. In all, 4 lots of KCl were coated using solutions containing 4%, 6%, 10% and 15% of Eudragit E 100. The coated samples were termed as C1, C2, C3 and C4 respectively.

### Evaluation of coated KCl

#### Assay

The drug content of coating compositions was determined by titrimetric assay (Indian pharmacopeia, 2014).The sample of coated KCl equivalent to 150 mg of KCl was crushed in a mortar and dissolved in 50 mL of distilled water. Further, 2 drops of potassium chromate indicator were added and titrated with the 0.1 N AgNO_3_. The endpoint was dark red brownish color. The assay was done in triplicate.

#### Entrapment efficiency

% Entrapment efficiency = [Drug added - unentrapped drug)/Drug added] *100.

Coated KCl, 1 g was added to 50 mL of pH 6.8 buffer and gently stirred for 2 min. The dispersion was filtered and filtrate was titrated with 0.1 N AgNO_3_ in similar manner as described in the Assay section above.

#### In vitro drug release studies

The in vitro drug release profiles of compositions C1-C4 each equivalent to 500 mg of KCl were determined using 900 mL of phosphate buffer pH 6.8 maintained at 37 ± 0.5 °C. The paddles of USP Type II apparatus (TDL 06 L, Electrolab, India)were stirred at 50 rpm and 5 mL aliquots were withdrawn at 5, 10, 15, 30 min intervals and the equal amount of fresh medium was replaced. The amount of drug released was determined by titrimetric assay wherein 5 ml of aliquot was made up to 50 mL with distilled water and the solution was titrated against 0.1 N AgNO_3_ using potassium chromate indicator. Similar study was performed on all the coated compositions by using 0.1 N hydrochloric acid (HCl) as a dissolution medium.

#### Particle size

Particle size of composition C3 was determined by microscopy technique (Labomed Lx 300 equipped with Image Pro Premier software, USA). The smear of aqueous dispersion of sample was observed under 10X magnification and size of particles from at least 20 fields was noted.

#### Flow properties

Composition C3 was subjected to determination of angle of repose and Hausner ratio by reported methods (Khar et al., 2013).

#### Microscopy

Pure KCl and the samples of composition C2 and C3 were observed under microscope (Labomed Lx 300, USA) for morphology. Photomicrographs of the fields were taken.

#### Scanning electron microscopy (SEM)

The surface characteristics of composition C2 and C3 were examined by means of a scanning electron microscope (Quanta-200, Thermo Fischer Scientific, USA). The double-sided carbon tape was placed on aluminum stab. The stab was dipped in the sample and with the help of air blower, loose particles were removed. The sample was coated with gold particles by using bio-radpolaran sputter coater. The sample was placed in an evacuated chamber and was scanned in a controlled pattern by electron beam. Images of plain KCl and that of the coated ones were compared with each other.

### Development and evaluation of dry syrup formulations

All the ingredients were passed through 30# sieve and weighed accurately (Table 2). Sucralose, cherry flavor, methyl paraben and propyl paraben were mixed together and the mixture was added to rest of the bulk by geometric mixing. All the ingredients were mixed thoroughly in a polythene bag for 5–7 min. The formulation was then filled in the glass bottles marked with 30 mL volume and stored at room temperature until further evaluation.

**Table 2:** Trials for the development of dry syrup formulation of coated KCl composition

Ingredients	Quantity in g							
	F1	F2	F3	F4	F5	F6	F7	F8
Composition C3^a^	6.6	6.6	6.6	6.6	6.6	6.6	6.6	6.6
Xanthan gum	0.3	**–**	**–**	**–**	**–**	**–**	**–**	**–**
HPMC K 100LV	**–**	0.3	**–**	**–**	**–**	**–**	**–**	**–**
Sodium carboxy methyl cellulose	**–**	**–**	0.3	**–**	**–**	**–**	**–**	**–**
Avicel CL 611	**–**	**–**	**–**	0.3	0.6	1.0	1.5	3
Sucralose	0.03	0.03	0.03	0.03	0.03	0.03	0.03	0.03
Disodium citro- phosphate	1.76	1.76	1.76	1.76	1.76	1.76	1.76	1.76
Citric acid	0.11	0.11	0.11	0.11	0.11	0.11	0.11	0.11
Cherry flavor	0.03	0.03	0.03	0.03	0.03	0.03	0.03	0.03
Methyl paraben	0.1	0.1	0.1	0.1	0.1	0.1	0.1	0.1
Propyl Paraben	0.01	0.01	0.01	0.01	0.01	0.01	0.01	0.01
Total	8.94	8.94	8.94	8.94	9.24	9.64	10.14	11.64

^a^Composition C3 is KCl coated with 10% w/w Eudragit E 100 solution

#### Organoleptic evaluation

The formulations were subjected to visual and olfactory examination.

#### Flow properties

Angle of repose and Hausner ratio of the dry syrup formulations were determined.

The dry syrup formulations were reconstituted by adding distilled water up to 30 mL mark in the bottle. The contents were mixed well by shaking, poured in a measuring cylinder and subjected to the following evaluation on day 1, 2, 3 and 7 of reconstitution.

#### Appearance

The reconstituted formulations were observed for appearance, colour and texture.

#### Redispersibility test

The redispersibility of the suspensions was checked by inverting the cylinder upside down until there was no sediment at the bottom of the cylinder.

#### Sedimentation volume

Suspension of each formulation was kept standing undisturbed at room temperature. The volume of sediment was noted on day 1, 2, 3 & 7 of reconstitution and the sedimentation ratio was calculated (Aulton, 2013).

#### Assay

The assay of reconstituted suspension of formula F8 was determined by titrimetry. About 1.5 mL of reconstituted suspension equivalent to 150 mg of KCl was triturated and dispersed in 50 mL distilled water. The suspension was titrated against 0.1 N AgNO_3_ using potassium chromate as an indicator until the sample turned reddish brown. The assay was done in triplicate. The assay was repeated on day 1, 2, 3 and 7 of reconstitution.

#### In vitro release studies

The in vitro drug release from formulation F8was studied separately in 0.1 N HCl and in pH 6.8 phosphate buffer in the manner similar to the one described in the evaluation of coated KCl compositions. The release studies were performed on day 1, 2, 3 and 7 of reconstitution.

Kesol syrup batch no KSL1644, (Mfg-Oct 2016- Exp-Sep 2019) was procured from the local pharmacy and subjected to in vitro drug release studies using 0.1 N HCl as a dissolution medium. The release profile of formulation F8 was compared with that of the marketed formulation.

#### Evaluation of taste using electronic taste sensing machine

Reconstituted suspension of formulation F8 and the marketed syrup formulation were coded as K1 and K2 respectively. At S. Zhaveri and Company, Mumbai, the formulations were subjected to the analysis of taste using Electronic taste sensing machine (TS-5000Z, Japan). Bitter sensor and salty sensor were used in the study. Reference electrode and sensor were initially dipped in a beaker containing a reference solution which comprised of 30 mM KCl and 0.3 mM tartaric acid. Potentiometric difference between each sensor and a reference electrode was measured and recorded by the E-tongue software (Linux) in terms of mV. For reference solution the output was termed as Vr. Similarly, membrane potentials Vs and Vt respectively for the marketed formulation and formulationF8 were obtained. The difference between sample and reference viz. Vs-Vr and Vt-Vr was compared (Keating et al., 2017; Latha & Lakshmi, 2012; Lorenz et al., 2009).

### Stability studies of optimized dry syrup formulation

Dry syrup formulation was prepared in bulk using the composition of formulation F8 (containing optimized concentration of suspending agent Avicel CL 611). The appropriate quantity was filled in each glass bottle, capped, sealed and subjected to accelerated storage conditions of 40 ± 2 °C/75 ± 5% RH for the period of three months. The samples were withdrawn at one, two and three-month intervals and subjected to all the tests mentioned in the section of evaluation of dry syrup formulations.

## Results

### Fluid bed coating of KCl with Eudragit E100

Compositions C1, C2, C3 and C4 of coated KCl were obtained by using 4, 6, 10 and 15% *w*/w Eudragit E100 solutions respectively. Though KCl is thermostable, the temperature of the fluid bed was kept below 45 °C to avoid exceeding glass transition temperature (Tg) of Eudragit E (Tg - 45-50 °C) (Malik et al., 2011). Compositions C1-C3 (Eudragit E100: 4–10%) did not encounter any problems during fluid bed processing whereas fluid bed processing of composition C4 (15% Eudragit E100) resulted in the sticking of powder particles to the walls of fluid bed processor which resulted in the decrease in fluidization and overall inefficient coating process.

### Evaluation of coated KCl

#### Assay, in vitro KCl release and micromeritics of compositions C1-C4

Assay of all the compositions of coated KCl was in the range of 98 to 101%. Entrapment efficiency for composition C1-C4 was found to be 69.2 ± 0.3, 74.5 ± 0.2, 81.1 ± 0.4 and 84 ± 0.4% respectively indicating greater encapsulation efficiency of KCl with increase in the level of Eudragit E100 concentration in the coating trials.

The in vitro release profiles of all the compositions are depicted in Table 3.

**Table 3:** In vitro release studies of various coated compositions of KCl

Time Min	%Cumulative KCl Release (0.1NHCl)				%Cumulative KCl Release (pH 6.8 phosphate buffer)			
	C1	C2	C3	C4	C1	C2	C3	C4
5	56.0 ± 1.3	51.9 ± 1.6	53.3 ± 4.5	42.8 ± 2.9	9.94 ± 2.2	8.94 ± 2.6	5.96 ± 1.5	3.47 ± 4.3
10	73.0 ± 1.3	79.1 ± 3.0	63.11 ± 2.3	58.4 ± 5.1	13.41 ± 3.5	11.43 ± 1.4	8.44 ± 2.3	5.96 ± 2.5
15	89.1 ± 2.2	87.2 ± 1.2	72.3 ± 3.6	65.2 ± 2.3	16.40 ± 4.4	14.91 ± 3.7	9.43 ± 3.0	6.95 ± 4.5
30	98.8 ± 2.1	92.6 ± 3.1	95.8 ± 2.9	86.9 ± 2.8	28.86 ± 2.4	23.90 ± 3.4	15.93 ± 2.5	9.46 ± 4.7

All the values are average ± standard deviation of *n* = 6

Compositions C1 and C2 showed more than 90% release in acidic medium within first 15 min as compared to composition C3 which showed almost complete release at the end of 30 min. Composition C4 could retard the release to a greater extent but had shown sticking problem during coating process.

The release studies of coated KCl were also done in pH 6.8 phosphate buffer (~ saliva pH). In case of compositions, C1 and C2, more than 20% release was observed at the end of 30 min whereas, compositions C3 and C4 (containing higher % of Eudragit E100) showed about 15 and 10% release respectively. This indicated better coating efficiency of composition C3 and C4 with lesser extent of drug release in saliva and thus better masking of the taste. Composition C3 was selected for the further studies as it showed optimum encapsulation, no issues in the fluid bed processing, absence of burst release in the stomach and saliva pH values.

Since the aim of the present work was to develop a dry syrup formulation, evaluation of micromeritic properties of the powder was important to understand the ease of handling and filling of the formulation. Composition C3 showed the average particle size of 316.2 ± 29.4 μm, angle of repose of 35^0^ and Hausner ratio of 1.2. Both the parameters indicated good flow characteristics of the composition (Khar et al., 2013). Too small particles may affect the flow of the powders while coarser particles would have unacceptable mouth-feel. Composition C3 with optimum particle size and passable flow properties was considered suitable for the formulation development.

#### Microscopic evaluation

**Fig. 1:**
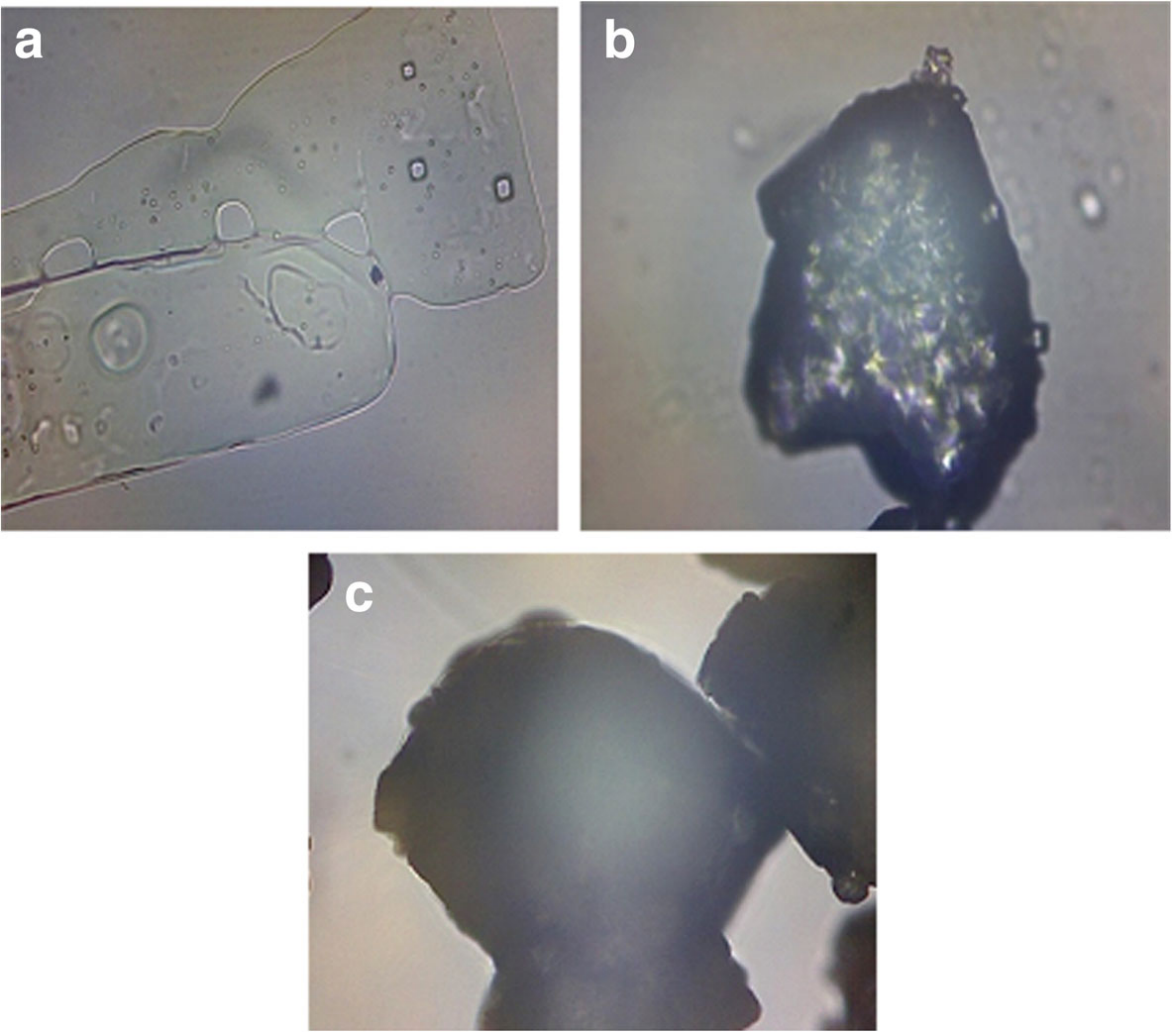
Photomicrographs of **a** Uncoated KCl **b** Composition C2 of KCl coated with 6% Eudragit E 100 solution **c** Composition C3 of KCl coated with 10% Eudragit E 100 solution

**Fig. 2:**
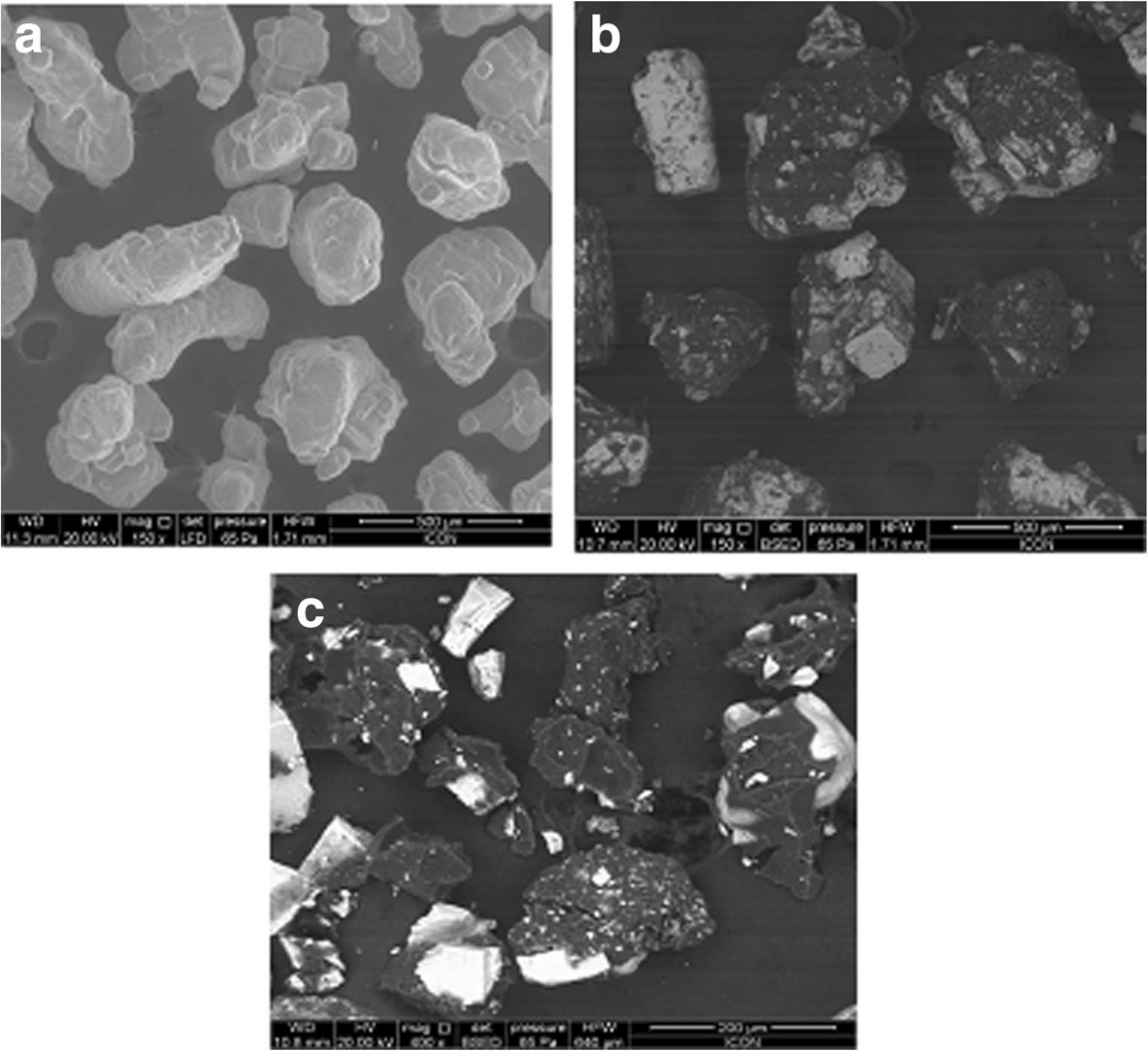
SEM images of **a** Uncoated KCl **b** Composition C2 of KCl coated with 6% Eudragit E 100 **c** Composition C3 of KCl coated with 10% Eudragit E 100 solution

The microscopic examination of uncoated KCl showed the presence of shiny and almost transparent crystals of KCl (Fig. 1a). Particles of composition C2 coated with 6% polymer solution showed opaque appearance interspersed with shiny spots indicating partially uncoated KCl (Fig. 1b) whereas particles of composition C3 coated with 10% solution showed uniform, opaque image with relatively lesser number of uncoated particles (Fig. 1c). SEM image of composition C3 distinctly showed dark coloured coating on the brighter drug particles (Fig. 2a) whereas the extent of coating was lesser in case of particles of composition C2 (Fig. 2b). Pure drug particles appear whitish with very irregular surface morphology (Fig. 2c). Microscopy studies thus indicated a direct proportion in the extent of coating with increasing concentration of Eudragit E100 in coating solution.

### Development and evaluation of dry syrup formulations

Formulation trials were taken using various suspending agents like xanthan gum, HPMC K100 LV, sodium carboxy methyl cellulose, and Avicel CL 611 (Table 2). Cherry flavor along with sucralose as a sweetener imparted pleasant organoleptic properties to the formulation. Disodium citrophosphate and citric acid maintained the formulation pH at 7 upon reconstitution. The resulting formulations in dry state and upon reconstitution were subjected to various evaluation parameters. Observations of these are given in Table 4.

**Table 4:** Evaluation of dry syrup formulations of composition C3 of coated KCl

	Dry form			Reconstituted form			
Formulation	Angle of repose (^0^)	Hausner ratio	Appearance	pH	Redispersibility	Sedimentation ratio	Assay^a^
F1	35.3	1.23	White –to -off white, phase separation	7.1	9	0.66	99.4 ± 0.2
F2	35.5	1.22	White –to -off white, homogeneous suspension	7.0	8	0.73	97.6 ± 0.6
F3	35.2	1.22	White –to- off white, homogeneous suspension	7.0	7	0.73	96.9 ± 0.8
F4	35.2	1.22	White –to -off white, homogeneous suspension	7.0	6	0.70	96.9 ± 0.7
F5	35.1	1.21	White –to -off white, phase separation	7.0	6	0.80	98.6 ± 0.4
F6	35	1.22	White –to -off white, homogeneous suspension	7.0	5	0.83	97.5 ± 0.1
F7	33.4	1.16	White –to- off white, homogeneous suspension	7.0	4	0.86	98.8 ± 0.8
F8	31.8	1.15	White –to -off white, homogeneous suspension	7.0	2	0.91	96.9 ± 0.5

^a^the assay values shown are average ± standard deviation of *n* = 3

Formulations F1 to F8 are the trial dry syrup formulations prepared with various suspending agents like F1 with 3.3% guar gum, F2 with 3.3% HPMC K 100LV, F3 with 3.3% sodium carboxymethyl cellulose, F4, F5, F6, F7 and F8 with 3.3%,6.5%,10%, 15% and 26%*w*/w Avicel CL 611 respectively.

#### In vitro release studies of reconstituted suspension

The results of drug release studies of formulation F8 on day 1, 2, 3 and 7 of reconstitution are given in Table 5.

**Table 5:** In vitro release studies of reconstituted formulation F8 on day 1, 2, 3 and 7 of reconstitution

% Cumulative release								
	0.1 N HCl				pH 6.8 Phosphate buffer^a^			
Time(min)	1st day	2nd day	3rd day	7th day	1st day	2nd day	3rd day	7th day
5	56.6 ± 0.3	57.1 ± 0.2	56.1 ± 0.2	73.0 ± 0.3	2.8 ± 0.5	5.6 ± 0.2	6.8 ± 0.9	10.3 ± 0.2
10	67.0 ± 0.8	66.4 ± 0.3	65.0 ± 0.5	82.9 ± 0.2	5.65 ± 0.2	6.9 ± 0.3	7.7 ± 0.2	12.3 ± 0.3
15	83.4 ± 0.3	80.5 ± 0.6	81.0 ± 0.3	96.9 ± 0.1	8.48 ± 0.4	8.5 ± 0.8	8.2 ± 0.2	15.8 ± 0.2
30	98.4 ± 0.1	95.4 ± 0.1	94.3 ± 0.2	99.8 ± 0.3	13.6 ± 2.9	13.8 ± 0.4	15.9 ± 2.6	20.3 ± 1.2

^a^lower drug release in pH 6.8 Phosphate buffer indicates better efficiency of coating of KCl with Eudragit E 100 and hence better taste masking. All the values are average ± standard deviation of *n* = 6

**Fig. 3:**
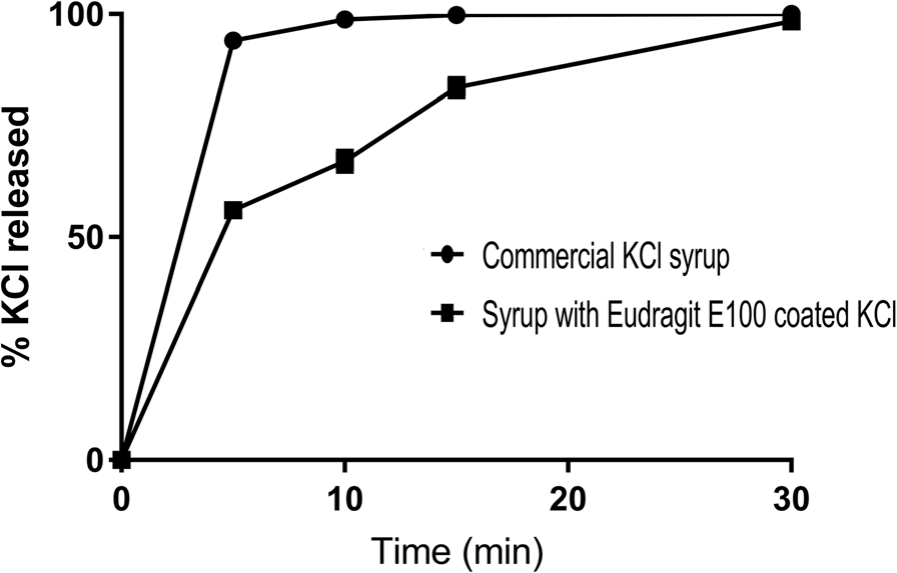
Comparison of*in vitro* dissolution profiles of marketed syrup formulation and reconstituted formulation F8. The observations are average ± standard deviation of *n* = 6 (The error bars are not visible in the figure owing to standard deviation values less than 2)

The graph showing comparison of drug release from formulation F8 in 0.1 N HCl on day 1 and that of marketed preparation is shown in (Fig. 3).

#### Taste evaluation using electronic taste sensing machine

During taste analysis, salty sensor gave better response than the bitter sensor. The software computed the sensory output in terms of change in the membrane potential of sensor as compared to the reference electrode. The difference between the potential shown by developed formulation and the reference solution was of 15 mV whereas that between the marketed formulation and reference solution was 32.5 mV which indicates that the taste was masked ~ 2-fold better in case of developed formulation as compared to the marketed formulation.

### Stability studies of optimized dry syrup formulation

Accelerated stability studies showed good physical stability of the formulation in dry as well reconstituted state. No significant difference was observed in assay and drug release profiles of stability samples (Table 6).

**Table 6:** Accelerated stability study of formulation F8

Sampling Interval	Parameters	Observations
1 month	Appearance	White–to- off white, smooth, suspension
	Taste on day 1,2,3,7	Pleasant
	pH on day 1,2,3,7	6.7–6.8
	Sedimentation volume	0.86
	Assay on day 1,2,3,7	99.5–99.1
2 month	Appearance	White–to- off white, smooth, suspension
	Taste on day 1,2,3,7	Pleasant
	pH on day 1,2,3,7	6.7–6.8
	Sedimentation volume	0.85
	Assay	97.4–96.9
3 month	Appearance	White–to -off white, smooth, suspension
	Taste on day 1,2,3,7	Pleasant
	pH on day 1,2,3,7	6.7–6.8
	Sedimentation volume	0.85
	Assay	96.4–96.2

Evaluation of Formulation F8 upon reconstitution at 1, 2 and 3 month intervals of storage at accelerated conditions of 40 ± 2 °C/75 ± 5% RH

## Discussion

Currently, KCl syrup is the only option available in India for paediatric and geriatric patients. The taste of this formulation is considered unacceptable clinically. While developing liquid oral formulations, taste masking of a drug is of paramount importance to enhance acceptance and compliance to the therapy by the patient (Faisal et al., 2018; Kaushik & Dureja, 2014). Depending on the extent of unacceptable taste, various approaches like addition of sweeteners, flavoring agents and viscosity builders (Thoke et al., 2012), complexation with cyclodextrins (Mahesh et al., 2010; Dinge & Nagarsenker, 2008; Prabhakaran et al., 2016), granulation (Pawar & Joshi, 2014), spray drying (Amelian et al., 2017a), ion exchange resins (Kouchak et al., 2018), hot melt extrusion (Pimparade et al., 2015) or conversion to a prodrug (Karaman, 2013) are employed for the taste-masking. Microencapsulation is one of the leading techniques for the taste-masking of a variety of drugs. Microencapsulation creates a physical barrier between the drug and the taste receptors in the form of a polymeric film thus leading to efficient taste masking. Although there are several ways to achieve microencapsulation, fluid bed processing is one of the most efficient, fast and economic techniques to achieve microcapsules with high yield, uniform coating and better entrapment efficiency leading to improved taste masking (Stange et al., 2014; Keser et al., 2018; Ghimire et al., 2018). In the present study, we sought to develop dry syrup formulation containing microencapsulated KCl which would not only mask the taste but also offer the advantages such as convenience of handling, lesser weight per container and overall better stability. We explored the potential of Eudragit E100 to coat and mask the taste of highly crystalline inorganic salts such as KCl which is hitherto not reported. We used commercially viable technique such as fluid bed coating to achieve Eudragit E100 coating. Eudragit E100 is a cationic amino alkyl methacrylate copolymer which solubilizes below pH 5.5 but remains insoluble at pH greater than 5.5. These solubility characteristics make it ideal for taste masking applications wherein the polymer would not dissolve in saliva (pH- 6.8 to 7.4) thus maintaining the physical barrier between the drug and the taste buds. Upon entering the stomach, it would dissolve in gastric fluids thus releasing the drug. It is safe for oral consumption and approved by the regulatory authorities (Rowe et al., 2009; Maniruzzaman et al., 2012). A number of reported studies have used this polymer for taste masking of various drugs by employing techniques like hot melt extrusion (Keating et al., 2017), spray-drying (Khan et al., 2007), spray congealing (Shah et al., 2008), granulation (Albertini et al., 2004), solvent evaporation (Douroumis et al., 2011) and fluid bed coating (Amelian et al., 2017b).

We carried out a series of investigations to evaluate a suitable amount of Eudragit E100 coating to obtain KCl particles with optimal characteristics for the development of palatable KCl dry syrup formulation. We found out that KCl coated with coating solution containing 10% Eudragit E100 (Composition C3 in Table 1) showed optimum KCl entrapment, good processability and micromeritics, and it did not lead to instantaneous release of KCl in buffers with pH values akin to the stomach as well as saliva. Hence, this was chosen for the further formulation development. Oral paediatric dose of KCl in the treatment of hypokalemia is 2-4 mEq/ kg/ day in divided doses (Potassium chloride label –FDA, 2017). The amount of coated KCl equivalent to 500 mg of pure KCl/5 mL was chosen as the dose for the development of dry syrup formulation.

Physical stability of a suspension formulation depends on various aspects like particle size of the dispersed phase, difference between the densities of the dispersed phase and dispersion medium as well as on the viscosity of the dispersion medium. Selection of a suitable suspending agent in optimum concentration is often the most practical approach for rendering the formulation physically stable. Various suspending agents viz. guar gum, HPMC K 100 LV, sodium carboxymethyl cellulose and Avicel CL 611were tried at equal concentration (3.3% *w*/w) in formulation F1, F2, F3 and F4 respectively. Guar gum requires longer time to wet and build the viscosity and hence formulation F1 upon reconstitution showed poor viscosity resulting in greater sedimentation rate. Upon standing for a day, the formulation acquired reasonable viscosity. The formulations F2 and F3 containing HPMC K 100 LV and sodium carboxymethyl cellulose showed higher sedimentation rate and poorer redispersibility compared to the formulation F4 which contained Avicel CL 611 as a suspending agent. Avicel CL 611 comprises 82–89% microcrystalline cellulose and 11–18% sodium carboxy methyl cellulose which offers the desired structured vehicle to the dosage form almost instantaneously upon reconstitution with water. To further improve the sedimentation ratio and redispersibility, formulations F5, F6, F7 and F8 were formulated with 6.5%, 10%, 15% and 26% w/w of Avicel CL 611 respectively. The sedimentation ratio increased linearly with the increment in the concentration of suspending agent and formulation F8 showed the ratio closest to unity upon keeping the suspension undisturbed over 7-day time period. The formulation redispersed homogeneously just in two inversions of the measuring cylinder holding the formulation. The overall appeal of the suspension formulation as well as the assurance of uniformity of the dosage depends on the sedimentation ratio and ease of redispersion. Sedimentation ratio closest to unity fulfils both the above criteria. Formulation F8 was thus considered as optimum and was taken up for the drug release, stability and electronic taste evaluation studies. As shown in Table 4, the in vitro release in 0.1 N HCl was more than 95% over the 30 min period. The release pattern did not change on 1st, 2nd, 3rd and 7th day of reconstitution. However, release in pH 6.8 buffer was found to increase (about 20% at the end of 30 min) on day 7 as compared to 13% on the day of reconstitution. This could be attributed to leaching of the drug from the polymer coating in presence of water. Marketed syrup formulation upon subjecting to dissolution studies in 0.1 N HCl showed complete dissolution of drug within 5 min unlike formulation F8 which could sustain the release for about 30 min.

Taste assessment using a multichannel taste sensor, an instrument commonly named as electronic tongue (e-Tongue), is an established alternative to human sensory analysis panel. A number of pharmaceutical laboratories around the world are using this instrument to assess the unacceptable taste of drugs and the masking efficiency of various techniques. In addition, it is used in placebo development, in taste matching of formulations, and in unknown-to-reference comparisons (Rachid et al., 2010).

In the present study, the formulation F8 and the marketed syrup formulation were compared for the taste using e-Tongue. The formulation F8 showed significantly better taste-masking of KCl compared to the marketed syrup formulation indicating the utility of Eudragit E100 coating to improve the palatability of oral KCl formulations.

## Conclusion

The dry syrup formulation developed using KCl coated with 10% Eudragit E 100 solution could effectively mask the unpleasant taste of the drug in comparison with marketed syrup formulation as confirmed by e-Tongue testing. The in vitro release of KCl could not be sustained beyond 30 min in acidic dissolution medium owing to limitations of using higher concentrations of Eudragit during fluid bed coating operation. Combination of Eudragit E 100 and a water insoluble polymer like ethyl cellulose could be tried in future to retard the drug release in gastric environment to effectively avoid gastric irritation and vomiting. The taste masked KCl particles could also find potential applications in food industry.

## Abbreviations

AgNO_3_: Silver nitrate
e-Tongue: Electronic tongue
HCl: Hydrochloric acid
KCl: Potassium chloride
SEM: Scanning Electron Microscopy
Tg: Glass transition temperature
